# Supplementary material for: Microbiome–host systems interactions: protective effects of propionate upon the blood–brain barrier
Source: Microbiome. 2018 Mar 21;6:55. doi: 10.1186/s40168-018-0439-y (PMC5863458; doi:10.1186/s40168-018-0439-y)
Supplement: Supplementary file 1 — Table S1. Effects of propionate treatment (1 μM, 24 h) upon mRNA expression of BBB-related genes in hCMEC/D3 cells, grouped in broad functional categories. Gene names listed in bold were significantly regulated compared to untreated cells (PFDR < 0.05). (PDF 381 kb) [file 40168_2018_439_MOESM1_ESM.pdf]

Cell Adhesion/Junctional proteins/Cytoskeletal factors

Symbol	Description	logFC	adj.P.Val
**PECAM1**	**platelet and endothelial cell adhesion molecule 1**	**-0.518**	**0.002**
**CLDN11**	**claudin 11**	**0.541**	**0.024**
GJA1	gap junction protein alpha 1	0.434	0.055
CLDN1	claudin 1	0.264	0.062
JAM3	junctional adhesion molecule 3	0.180	0.132
UTRN	utrophin	-0.155	0.157
CDH2	cadherin 2	0.167	0.184
CLDN7	claudin 7	-0.122	0.270
ANXA1	annexin A1	0.145	0.270
TJP2	tight junction protein 2	-0.124	0.281
CLDN17	claudin 17	-0.124	0.282
CLDN4	claudin 4	0.141	0.341
SMARCA2	SWI/SNF related, matrix associated, actin dependent regulator of chromatin, subfamily a, member 2	0.118	0.373
CLDN23	claudin 23	-0.108	0.462
JAM2	junctional adhesion molecule 2	-0.110	0.540
TJP1	tight junction protein 1	0.090	0.573
CLDN6	claudin 6	0.092	0.582
LAMA4	laminin subunit alpha 4	-0.078	0.629
LAMA3	laminin subunit alpha 3	-0.070	0.657
DAG1	dystroglycan 1	-0.067	0.663
CLDN20	claudin 20	0.073	0.666
AGRN	agrin	0.055	0.694
CLDN12	claudin 12	0.064	0.751
CLDN8	claudin 8	-0.049	0.753
CLDN15	claudin 15	-0.050	0.778
CTNNB1	catenin beta 1	-0.047	0.783
VIM	vimentin	0.041	0.792
HAPLN2	hyaluronan and proteoglycan link protein 2	-0.054	0.794
DTNA	dystrobrevin alpha	0.053	0.796
ESAM	endothelial cell adhesion molecule	-0.043	0.799
LAMB2	laminin subunit beta 2	-0.044	0.803
CLDN9	claudin 9	-0.039	0.804
LAMA2	laminin subunit alpha 2	-0.057	0.808
ITM2A	integral membrane protein 2A	-0.041	0.837
FN1	fibronectin 1	-0.037	0.852
COL4A1	collagen type IV alpha 1 chain	0.030	0.875
TJP3	tight junction protein 3	-0.027	0.894
CLDN3	claudin 3	-0.023	0.906
GJB6	gap junction protein beta 6	0.022	0.911
CDH5	cadherin 5	-0.029	0.920
LAMA1	laminin subunit alpha 1	0.014	0.948
CLDN5	claudin 5	-0.016	0.962
CLDN22	claudin 22	0.013	0.963
ACTB	actin beta	0.009	0.965
CLDN10	claudin 10	-0.009	0.966
ADGRA2	adhesion G protein-coupled receptor A2	0.010	0.966
ITGA3	integrin subunit alpha 3	-0.009	0.967

OCLN	occludin	-0.007	0.969
HSPG2	heparan sulfate proteoglycan 2	0.008	0.973
DMD	dystrophin	-0.003	0.989
AFDN	afadin, adherens junction formation factor	0.003	0.989
MARVELD2	MARVEL domain containing 2	-0.001	0.996

Transporter proteins

Symbol	Description	logFC	adj.P.Val
**SLC1A5**	**solute carrier family 1 member 5**	**0.400**	**0.011**
**SLC44A1**	**solute carrier family 44 member 1**	**-0.261**	**0.030**
SLC7A5	solute carrier family 7 member 5	0.206	0.092
TFRC	transferrin receptor	0.262	0.099
SLC38A5	solute carrier family 38 member 5	0.194	0.165
SLC38A3	solute carrier family 38 member 3	0.140	0.240
SLC22A5	solute carrier family 22 member 5	0.140	0.272
SLC29A4	solute carrier family 29 member 4	0.144	0.299
SLC22A8	solute carrier family 22 member 8	-0.126	0.308
SLC2A1	solute carrier family 2 member 1	-0.129	0.342
SLC38A2	solute carrier family 38 member 2	0.120	0.381
SLC28A2	solute carrier family 28 member 2	0.133	0.411
SLC5A1	solute carrier family 5 member 1	0.100	0.447
SLC5A6	solute carrier family 5 member 6	0.094	0.452
SLC6A6	solute carrier family 6 member 6	0.096	0.461
SLC1A4	solute carrier family 1 member 4	-0.115	0.462
SLC27A4	solute carrier family 27 member 4	-0.139	0.463
LRP2	LDL receptor related protein 2	0.082	0.501
SLC38A1	solute carrier family 38 member 1	0.091	0.510
SLC22A1	solute carrier family 22 member 1	0.078	0.560
LDLR	low density lipoprotein receptor	-0.075	0.566
SLC1A3	solute carrier family 1 member 3	0.084	0.581
MFSD2A	major facilitator superfamily domain containing 2A	0.079	0.593
ABCG2	ATP binding cassette subfamily G member 2 (Junior blood group)	0.066	0.671
INSR	insulin receptor	0.060	0.718
AQP4	aquaporin 4	0.060	0.733
SLC16A2	solute carrier family 16 member 2	-0.057	0.780
ABCC5	ATP binding cassette subfamily C member 5	-0.041	0.793
SLCO1C1	solute carrier organic anion transporter family member 1C1	0.041	0.795
SLC29A1	solute carrier family 29 member 1 (Augustine blood group)	0.036	0.807
SLC27A1	solute carrier family 27 member 1	-0.036	0.818
SLC7A3	solute carrier family 7 member 3	0.038	0.824
SLC22A2	solute carrier family 22 member 2	0.035	0.843
SLC16A1	solute carrier family 16 member 1	-0.047	0.847
ABCB1	ATP binding cassette subfamily B member 1	0.029	0.866
AGER	advanced glycosylation end-product specific receptor	-0.026	0.908
AVPR1A	arginine vasopressin receptor 1A	-0.023	0.912
ABCA2	ATP binding cassette subfamily A member 2	0.015	0.947
SLC6A9	solute carrier family 6 member 9	0.013	0.949
SLC1A1	solute carrier family 1 member 1	-0.013	0.954
SLC7A1	solute carrier family 7 member 1	0.013	0.955
ABCC1	ATP binding cassette subfamily C member 1	0.012	0.956

SLC22A3	solute carrier family 22 member 3	-0.012	0.957
LEPR	leptin receptor	-0.009	0.960
SLC16A7	solute carrier family 16 member 7	-0.012	0.962
ABCC4	ATP binding cassette subfamily C member 4	-0.011	0.963
SLC5A3	solute carrier family 5 member 3	-0.009	0.967
SLC7A6	solute carrier family 7 member 6	-0.008	0.969
SLCO2B1	solute carrier organic anion transporter family member 2B1	-0.005	0.983
ABCC2	ATP binding cassette subfamily C member 2	-0.003	0.988
SLCO1B1	solute carrier organic anion transporter family member 1B1	0.003	0.988
SLC2A13	solute carrier family 2 member 13	0.003	0.990
SLC1A2	solute carrier family 1 member 2	0.001	0.995

Inflammatory response

Symbol	Description	logFC	adj.P.Val
**TNFSF10**	**tumor necrosis factor superfamily member 10**	**-0.684**	**0.001**
**PDGFRB**	**platelet derived growth factor receptor beta**	**-0.441**	**0.015**
**TNFRSF1A**	**TNF receptor superfamily member 1A**	**-0.289**	**0.021**
**TNFRSF12A**	**TNF receptor superfamily member 12A**	**0.383**	**0.028**
**TNFRSF21**	**TNF receptor superfamily member 21**	**0.325**	**0.031**
ITGB4	integrin subunit beta 4	-0.205	0.056
TNFAIP6	TNF alpha induced protein 6	0.325	0.118
PODXL	podocalyxin like	-0.194	0.130
ITGA5	integrin subunit alpha 5	-0.211	0.163
ITGA1	integrin subunit alpha 1	-0.150	0.188
PTGS2	prostaglandin-endoperoxide synthase 2	0.187	0.189
ITGB5	integrin subunit beta 5	-0.156	0.193
CXCL2	C-X-C motif chemokine ligand 2	0.171	0.231
IKBKB	inhibitor of nuclear factor kappa B kinase subunit beta	-0.139	0.299
SOD1	superoxide dismutase 1, soluble	0.126	0.338
ITGB8	integrin subunit beta 8	-0.144	0.340
NOS1	nitric oxide synthase 1	0.114	0.366
CCR5	C-C motif chemokine receptor 5 (gene/pseudogene)	0.222	0.391
ITGA4	integrin subunit alpha 4	0.161	0.430
CLEC5A	C-type lectin domain family 5 member A	0.138	0.441
ITGA6	integrin subunit alpha 6	-0.092	0.442
GRN	granulin precursor	-0.089	0.455
MMP9	matrix metallopeptidase 9	-0.099	0.475
NR3C1	nuclear receptor subfamily 3 group C member 1	-0.085	0.496
CRH	corticotropin releasing hormone	-0.092	0.558
AGT	angiotensinogen	-0.091	0.594
PTGDS	prostaglandin D2 synthase	-0.097	0.596
NOX4	NADPH oxidase 4	0.070	0.601
MMP2	matrix metallopeptidase 2	-0.088	0.687
SELP	selectin P	-0.074	0.689
IL1RN	interleukin 1 receptor antagonist	0.060	0.692
CXCR3	C-X-C motif chemokine receptor 3	-0.060	0.711
F11R	F11 receptor	-0.089	0.741
TNFRSF1B	TNF receptor superfamily member 1B	-0.098	0.760
SEMA7A	semaphorin 7A (John Milton Hagen blood group)	0.054	0.770
ITGB3	integrin subunit beta 3	0.049	0.793

ITGAV	integrin subunit alpha V	-0.033	0.837
TLR2	toll like receptor 2	0.030	0.860
ITGB1	integrin subunit beta 1	0.026	0.880
PTGER3	prostaglandin E receptor 3	-0.022	0.895
TNF	tumor necrosis factor	-0.016	0.927
ITGB2	integrin subunit beta 2	-0.012	0.951
IL1B	interleukin 1 beta	-0.033	0.967
CCR2	C-C motif chemokine receptor 2	-0.007	0.970
CD276	CD276 molecule	0.006	0.973
C3	complement C3	-0.001	0.997

Vascular function/coagulation cascade

Symbol	Description	logFC	adj.P.Val
**SERPINE2**	**serpin family E member 2**	**0.461**	**0.007**
**PROCR**	**protein C receptor**	**0.240**	**0.046**
PLAT	plasminogen activator, tissue type	-0.242	0.051
SERPINE1	serpin family E member 1	0.244	0.212
PROS1	protein S (alpha)	-0.165	0.262
PROC	protein C, inactivator of coagulation factors Va and VIIIa	-0.128	0.479
CA1	carbonic anhydrase 1	0.081	0.518
VWF	von Willebrand factor	-0.139	0.567
AVP	arginine vasopressin	-0.057	0.758
SERPINI1	serpin family I member 1	-0.033	0.840
PLG	plasminogen	-0.030	0.884
KNG1	kininogen 1	-0.024	0.898
NOS3	nitric oxide synthase 3	0.037	0.905
MYLK	myosin light chain kinase	-0.014	0.949
PTAFR	platelet activating factor receptor	-0.013	0.952
EPAS1	endothelial PAS domain protein 1	0.010	0.955

Endothelial proliferation/angiogenesis

Symbol	Description	logFC	adj.P.Val
PDGFB	platelet derived growth factor subunit B	-0.226	0.090
TMEFF2	transmembrane protein with EGF like and two follistatin like domains 2	0.166	0.170
S100A12	S100 calcium binding protein A12	0.143	0.245
FGF19	fibroblast growth factor 19	-0.157	0.354
IGFBP3	insulin like growth factor binding protein 3	0.091	0.486
RGS5	regulator of G-protein signaling 5	-0.075	0.548
FLT1	fms related tyrosine kinase 1	-0.112	0.572
HNRNPDL	heterogeneous nuclear ribonucleoprotein D like	-0.084	0.601
VEGFA	vascular endothelial growth factor A	-0.072	0.617
S100B	S100 calcium binding protein B	-0.071	0.643
EZH1	enhancer of zeste 1 polycomb repressive complex 2 subunit	-0.068	0.722
PTPRB	protein tyrosine phosphatase, receptor type B	-0.057	0.745
HMGB1	high mobility group box 1	0.044	0.775
PTN	pleiotrophin	-0.029	0.920
KDR	kinase insert domain receptor	0.022	0.934
BTG2	BTG anti-proliferation factor 2	0.012	0.958
EPO	erythropoietin	-0.011	0.963

Other BBB-related genes

Symbol	Description	logFC	adj.P.Val
EPHA2	EPH receptor A2	-0.249	0.076
MOG	myelin oligodendrocyte glycoprotein	-0.090	0.546
CLN3	CLN3, battenin	-0.085	0.566
SRGN	serglycin	0.072	0.574
MBP	myelin basic protein	-0.066	0.646
RAMP2	receptor activity modifying protein 2	0.054	0.713
CLCN2	chloride voltage-gated channel 2	-0.055	0.733
CPE	carboxypeptidase E	0.044	0.811
CYBB	cytochrome b-245 beta chain	0.033	0.856
MPZL1	myelin protein zero like 1	0.028	0.864
GAB2	GRB2 associated binding protein 2	-0.030	0.866
MAP3K7	mitogen-activated protein kinase kinase kinase 7	0.028	0.882
APP	amyloid beta precursor protein	-0.044	0.890
APLP2	amyloid beta precursor like protein 2	0.022	0.914
PLP1	proteolipid protein 1	0.019	0.935
HDC	histidine decarboxylase	0.007	0.985
HRH3	histamine receptor H3	0.003	0.989
APOE	apolipoprotein E	0.000	1.000
GFAP	glial fibrillary acidic protein	0.000	1.000
