# Supplementary figures and images for: Microbiome–host systems interactions: protective effects of propionate upon the blood–brain barrier
Source: Microbiome. 2018 Mar 21;6:55. doi: 10.1186/s40168-018-0439-y (PMC5863458; doi:10.1186/s40168-018-0439-y)

Figure S1

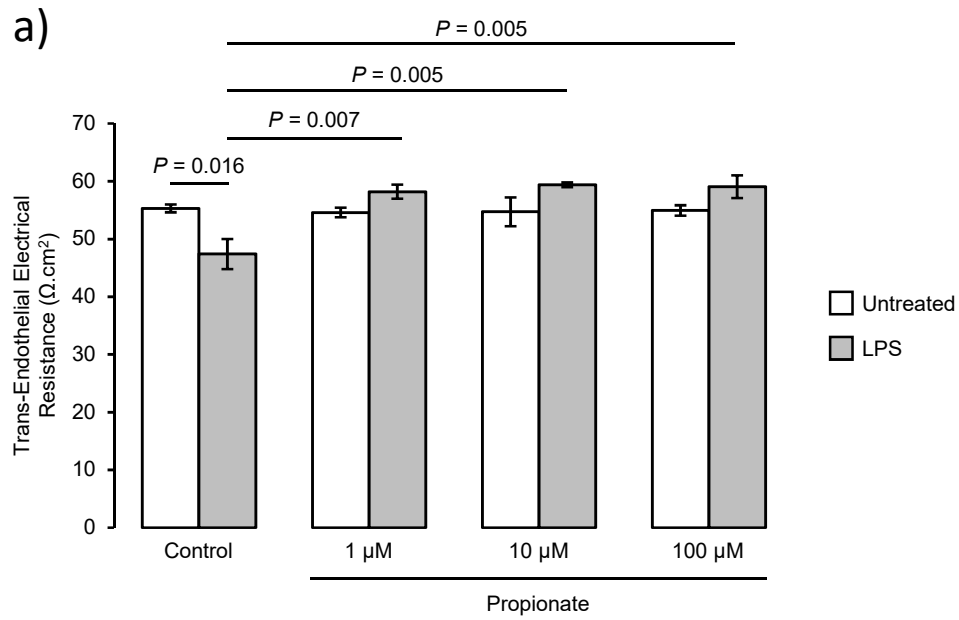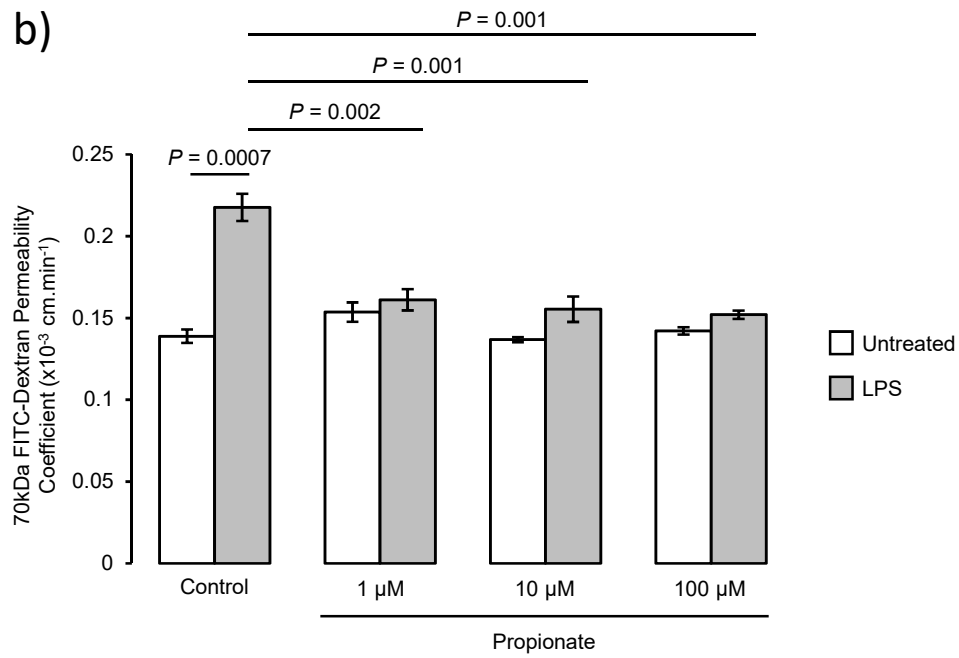

Supplement: Supplementary file 2 — Figure S1. Persistence of the protective effect of propionate upon LPS-induced barrier disruption across different doses. (a) Assessment of the paracellular permeability of hCMEC/D3 monolayers to 70 kDa FITC–dextran following treatment for 24 h with 1, 10 or 100 μM propionate, with or without inclusion of 50 ng/ml LPS for the last 12 h of incubation; data are mean ± SEM, n = 3 independent experiments. (b) Trans-endothelial electrical resistance of hCMEC/D3 monolayers following treatment for 24 h with 1, 10 or 100 μM propionate, with or without inclusion of 50 ng/ml LPS for the last 12 h of incubation; data are mean ± SEM, n = 3 independent experiments. (PDF 341 kb) [file 40168_2018_439_MOESM2_ESM.pdf]

Figure S2

a)

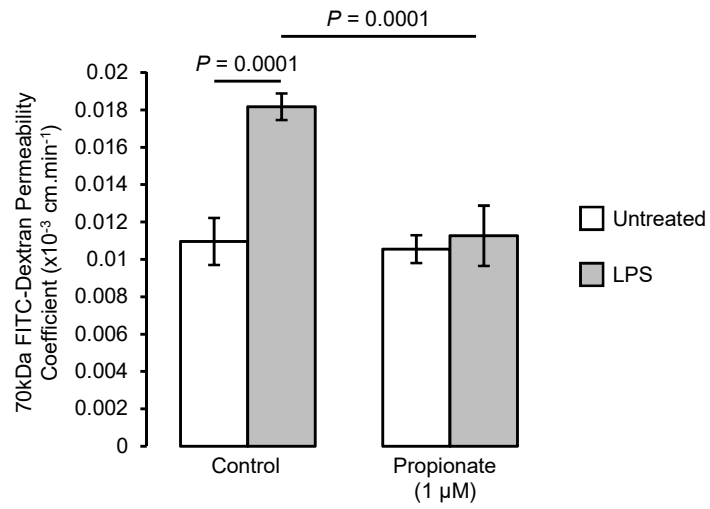

b)

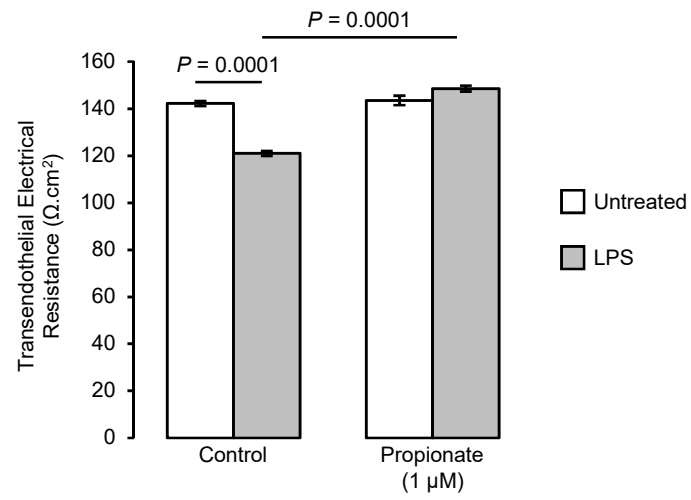

Supplement: Supplementary file 3 — Figure S2. Protective effects of propionate against LPS-induced barrier disruption in primary human brain microvascular endothelial cells (HBMEC). (a) Assessment of the paracellular permeability of HBMEC monolayers to 70 kDa FITC–dextran following treatment for 24 h with 1 μM propionate, with or without inclusion of 50 ng/ml LPS for the last 12 h of incubation; data are mean ± SEM, n = 3 independent experiments. (b) Trans-endothelial electrical resistance of HBMEC monolayers following treatment for 24 h with 1 μM propionate, with or without inclusion of 50 ng/ml LPS for the last 12 h of incubation; data are mean ± SEM, n = 3 independent experiments. (PDF 321 kb) [file 40168_2018_439_MOESM3_ESM.pdf]

Figure S3

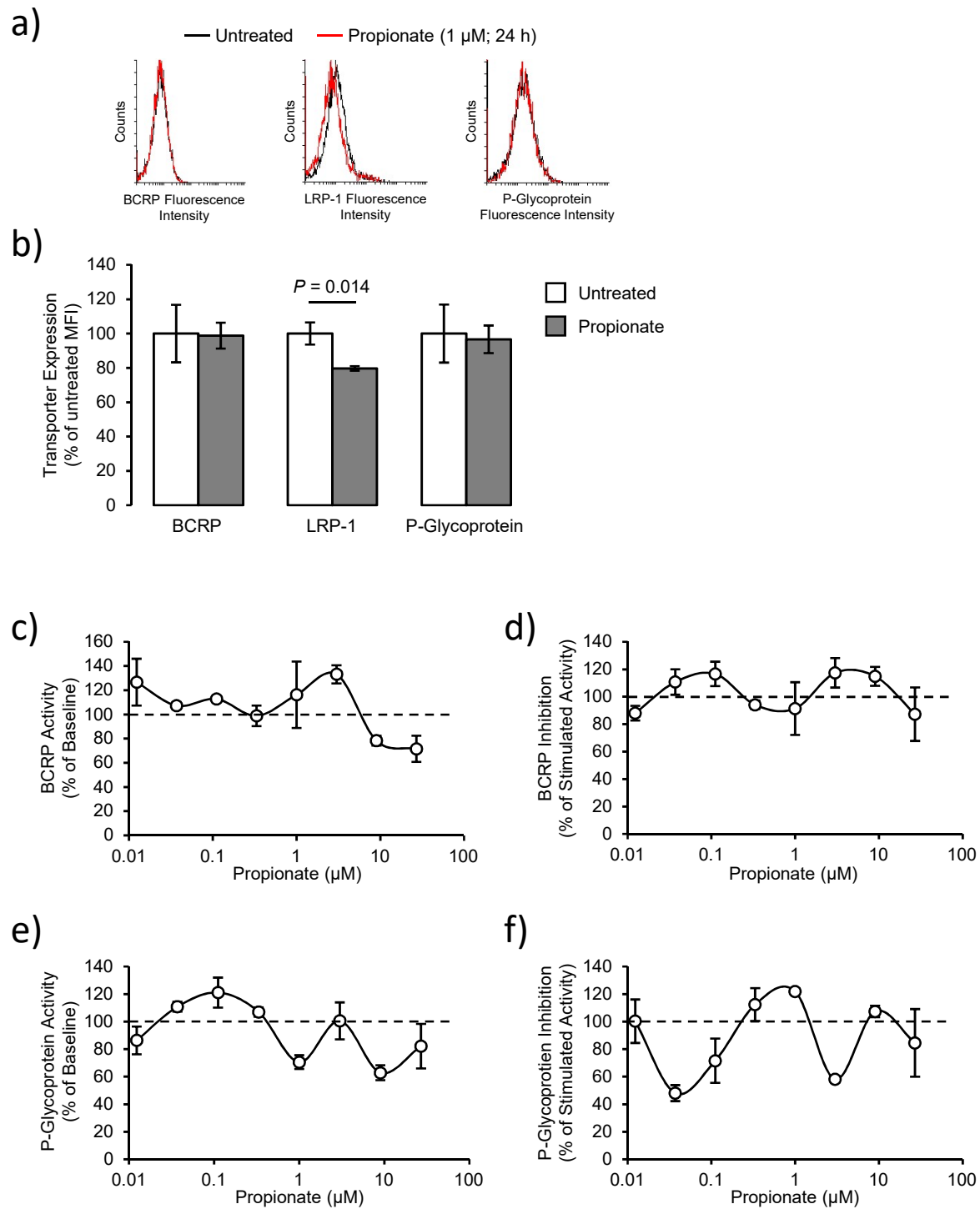

Supplement: Supplementary file 5 — Figure S3. Effects of propionate upon expression and activity of typical cerebromicrovascular efflux transporter systems. (a) Surface expression of BCRP, LRP-1 and P-glycoprotein on control and propionate-treated (1 μM, 24 h) hCMEC/D3 cells (black, control, red, propionate), data are representative of three independent experiments. (b) Median fluorescence intensity of surface expression of BCRP, LRP-1 and P-glycoprotein on control and propionate-treated (1 μM, 24 h) hCMEC/D3 cells; data are mean ± SEM, n = 3 independent experiments. (c) Lack of stimulatory effect of propionate upon BCRP, data are mean ± SEM, n = 4. (d) Lack of inhibitory effect of propionate upon stimulated ATP-dependent activity of BCRP, data are mean ± SEM, n = 4. (e) Lack of stimulatory effect of propionate upon P-glycoprotein, data are mean ± SEM, n = 4. (f) Lack of inhibitory effect of propionate upon stimulated ATP-dependent activity of P-glycoprotein, data are mean ± SEM, n = 4. (PDF 437 kb) [file 40168_2018_439_MOESM5_ESM.pdf]
