# Supplementary material for: Microbiome–host systems interactions: protective effects of propionate upon the blood–brain barrier
Source: Microbiome. 2018 Mar 21;6:55. doi: 10.1186/s40168-018-0439-y (PMC5863458; doi:10.1186/s40168-018-0439-y)
Supplement: Supplementary file 4 — Table S2. Effects of propionate treatment (1 μM, 24 h) upon mRNA expression of antioxidant system-related genes in hCMEC/D3 cells. Gene names listed in bold were significantly regulated compared to untreated cells (PFDR < 0.05). (PDF 385 kb) [file 40168_2018_439_MOESM4_ESM.pdf]

Table S2: Human anti-oxidant genes included in array analyses in this study

Symbol	log_2 fold change	Adjusted *P* value	Synonyms	Description
**GCLM†**	**1.034**	**1.312×10^{-4}**	**GLCLR**	**glutamate-cysteine ligase modifier subunit**
**SRXN1**	**1.242**	**1.934×10^{-4}**	**C20orf139, Npn3, SRX, SRX1**	**sulfiredoxin 1**
**TXNRD1***	**0.928**	**2.770×10^{-4}**	**GRIM-12, TR, TR1, TRXR1, TXNR**	**thioredoxin reductase 1**
**HMOX1†**	**0.693**	**2.770×10^{-4}**	**HMOX1D, HO-1, HSP32, bK286B10**	**heme oxygenase 1**
**FTL†**	**0.564**	**2.467×10^{-3}**	**LFTD, NBIA3**	**ferritin light chain**
**SLC7A11†**	**0.649**	**3.676×10^{-3}**	**CCBR1, xCT**	**solute carrier family 7 member 11**
**TXNL4B**	**0.388**	**6.601×10^{-3}**	**DLP, Dim2**	**thioredoxin like 4B**
**NQO1†**	**0.345**	**0.015**	**DHQU, DIA4, DTD, NMOR1, NMORI, QR1**	**NAD(P)H quinone dehydrogenase 1**
**TXNDC9**	**0.346**	**0.020**	**APACD, PHLP3**	**thioredoxin domain containing 9**
**PRDX1***	**0.306**	**0.021**	**MSP23, NKEF-A, NKEFA, PAG, PAGA, PAGB, PRX1, PRXI, TDPX2**	**peroxiredoxin 1**
**MT1F**	**-0.254**	**0.039**	**MT1**	**metallothionein 1F**
**MT1G**	**0.224**	**0.043**	**MT1, MT1K**	**metallothionein 1G**
**GLRX3**	**0.256**	**0.045**	**GLRX4, GRX3, GRX4, PICOT, TXNL2, TXNL3**	**glutaredoxin 3**
**TXN***	**0.240**	**0.050**	**TRDX, TRX, TRX1**	**thioredoxin**
FTH1†	0.206	0.061	FHC, FTH, FTHL6, HFE5, PIG15, PLIF	ferritin heavy chain 1
GSR*	0.328	0.061	HEL-75, HEL-S-122m	glutathione-disulfide reductase
MSRA	-0.222	0.082	PMSR	methionine sulfoxide reductase A
TXNDC5	0.217	0.085	ENDOPDI, ERP46, HCC-2, HCC2, PDIA15, STRF8, UNQ364	thioredoxin domain containing 5
MT1M	-0.213	0.106	MT-1M, MT-IM, MT1, MT1K	metallothionein 1M
GPX7	0.210	0.110	CL683, GPX6, GPx-7, GSHPx-7, NPGPx	glutathione peroxidase 7
TXNRD2	-0.166	0.164	SELZ, TR, TR-BETA, TR3, TRXR2	thioredoxin reductase 2
ERP44	0.170	0.165	PDIA10, TXNDC4	endoplasmic reticulum protein 44
PRDX4	0.153	0.173	AOE37-2, AOE372, HEL-S-97n, PRX-4	peroxiredoxin 4
SOD2	0.191	0.238	IPO-B, IPOB, MNSOD, MVCD6, Mn-SOD	superoxide dismutase 2, mitochondrial
PDIA6	0.132	0.250	ERP5, P5, TXNDC7	protein disulfide isomerase family A member 6
TXNDC8	-0.176	0.262	SPTRX-3, TRX6, bA427L11.2	thioredoxin domain containing 8
GPX4	-0.125	0.315	GPx-4, GSHPx-4, MCSP, PHGPx, SMDS, snGPx, snPHGPx	glutathione peroxidase 4
SOD1	0.126	0.338	ALS, ALS1, HEL-S-44, IPOA, SOD, hSod1, homodimer	superoxide dismutase 1, soluble

Symbol	log_2 fold change	Adjusted *P* value	Synonyms	Description
TMX1	0.140	0.354	PDIA11, TMX, TXNDC, TXNDC1	thioredoxin related transmembrane protein 1
GLRX	-0.110	0.375	GRX, GRX1	glutaredoxin
TXNDC17	0.111	0.404	TRP14, TXNL5	thioredoxin domain containing 17
MT1A	-0.096	0.479	MT1, MT1S, MTC	metallothionein 1A
PRDX3	0.084	0.486	AOP-1, AOP1, HBC189, MER5, PRO1748, SP-22, prx-III	peroxiredoxin 3
GLRX2	0.086	0.500	CGI-133, GRX2	glutaredoxin 2
NME9	0.093	0.506	NM23-H9, TXL-2, TXL2, TXNDC6	NME/NM23 family member 9
TXNDC12	0.114	0.539	AG1, AGR1, ERP16, ERP18, ERP19, PDIA16, TLP19, hAG-1, hTLP19	thioredoxin domain containing 12
MT1X	0.104	0.544	MT-1l, MT1	metallothionein 1X
PRDX6	0.081	0.549	1-Cys, AOP2, HEL-S-128m, NSGPx, PRX, aiPLA2, p29	peroxiredoxin 6
GPX6	-0.087	0.567	GPX5p, GPXP3, GPx-6, GSHPx-6, dJ1186N24, dJ1186N24.1	glutathione peroxidase 6
GPX3	-0.073	0.622	GPx-P, GSHPx-3, GSHPx-P	glutathione peroxidase 3
TMX2	0.063	0.637	CGI-31, PDIA12, PIG26, TXNDC14	thioredoxin related transmembrane protein 2
CAT	0.068	0.642	-	catalase
PRDX5	-0.064	0.662	ACR1, AOEB166, B166, HEL-S-55, PLP, PMP20, PRDX6, PRXV, SBBI10, prx-V	peroxiredoxin 5
MT1E	-0.091	0.687	MT-1E, MT-IE, MT1, MTD	metallothionein 1E
GPX5	0.053	0.694	HEL-S-75p	glutathione peroxidase 5
GPX1	-0.059	0.698	GPXD, GSHPX1	glutathione peroxidase 1
SOD3	-0.048	0.763	EC-SOD	superoxide dismutase 3, extracellular
GLRX5	-0.047	0.777	C14orf87, FLB4739, GRX5, PR01238, PRO1238, PRSA, SIDBA3, SPAHGC	glutaredoxin 5
SELENOP	0.040	0.794	SELP, SEPP, SEPP1, SeP	selenoprotein P
TXN2	0.052	0.832	COXPD29, MT-TRX, MTRX, TRX2	thioredoxin 2
CCS	-0.031	0.841	-	copper chaperone for superoxide dismutase
MT1H	0.041	0.853	MT-0, MT-1H, MT-IH, MT1	metallothionein 1H
CP	0.023	0.889	CP-2	ceruloplasmin
TXNIP	0.151	0.899	ARRDC6, EST01027, HHCPA78, THIF, VDUP1	thioredoxin interacting protein
TXNDC11	-0.021	0.907	EFP1	thioredoxin domain containing 11
PRDX2	0.016	0.935	HEL-S-2a, NKEF-B, NKEFB, PRP, PRX2, PRXII, PTX1, TDPX1, TPX1, TSA	peroxiredoxin 2
MT2A	-0.014	0.942	MT2	metallothionein 2A
NME8	-0.015	0.943	CILD6, HEL-S-99, NM23-H8, SPTRX2, TXNDC3, sptrx-2	NME/NM23 family member 8
TXNDC2	-0.012	0.953	SPTRX, SPTRX1	thioredoxin domain containing 2

Symbol	log_2 fold change	Adjusted *P* value	Synonyms	Description
TMX4	-0.011	0.961	DJ971N18.2, PDIA14, TXNDC13	thioredoxin related transmembrane protein 4
MT1B	-0.009	0.963	MT-1B, MT-IB, MT1, MT1Q, MTP	metallothionein 1B
TXNL1	-0.013	0.968	HEL-S-114, TRP32, TXL-1, TXNL, Txl	thioredoxin like 1
GPX2	-0.008	0.969	GI-GPx, GPRP, GPRP-2, GPx-2, GPx-GI, GSHPX-GI, GSHPx-2	glutathione peroxidase 2
TMX3	-0.007	0.982	PDIA13, TXNDC10	thioredoxin related transmembrane protein 3

†Anti-oxidant genes identified from Enrichr search and Gorrini *et al.* (2013), and included in **Fig. 3a**.

*Anti-oxidant genes identified from Enrichr search, Gorrini *et al.* (2013) and Gelain *et al.* (2009), and included in **Fig. 3a**.
